# Biological Plausibility Between Long-COVID and Periodontal Disease Development or Progression

**DOI:** 10.3390/biomedicines13123023

**Published:** 2025-12-10

**Authors:** Oelisoa Mireille Andriankaja, Sidney Whiteheart, Marcelo Barbosa de Accioly Mattos

**Affiliations:** 1 Center for Oral Health Research, College of Dentistry, University of Kentucky, Lexington, KY 40508, USA; oelisoa.andriankaja@uky.edu; 2 College of Medicine, University of Kentucky, Lexington, KY 40506, USA; whitehe@uky.edu; 3 College of Dentistry, University of Kentucky, Lexington, KY 40508, USA

**Keywords:** COVID-19, Long COVID, SARS-CoV-2, periodontitis, immunity, inflammation, oral microbiome, virus, risk factor, Epstein–Barr

## Abstract

**Background**: Long COVID (LC) is a multi-system disorder with persistent symptoms following SARS-CoV-2 infection. The presence of SARS-CoV-2 in the oral cavity and periodontium raises questions about its potential impact on periodontal health. **Methods**: A comprehensive literature search was conducted in PubMed using terms related to LC (e.g., “long-COVID,” “post-acute sequelae of SARS-CoV-2 infection,” “PASC,” “post-COVID-19,” “long-haul COVID”) and oral/periodontal diseases (e.g., “periodontal disease,” “periodontitis,” “gingiva,” “oral disease,” “dental”), filtered for English-language full-text articles published from 2019 to 2024. The search yielded 260 articles, which were supplemented with targeted searches on pathogenesis, immune mechanisms, microbiome alterations, and clinical outcomes, resulting in approximately 248 studies included in this review. **Results**: LC exhibits systemic immunoinflammatory dysregulation, including neutrophil activation, elevated pro-inflammatory cytokines, and complement activation, overlapping with mechanisms implicated in periodontitis. LC also leads to gastrointestinal and pulmonary dysbiosis, with potential effects on oral microbial communities. Gingival epithelium and periodontal ligament cells express ACE2, which is increased in periodontitis, facilitating viral entry. LC has been associated with reactivation of herpesviruses, such as Epstein–Barr virus, which are linked to autoimmune disorders and periodontitis. **Conclusions**: LC may act as a systemic risk factor for periodontitis. This review provides the theoretical foundation for the interactions between LC and oral health and highlights priorities for future epidemiologic and mechanistic research to better understand these relationships.

## 1. Introduction

As of 28 September 2024, Severe Acute Respiratory Syndrome Coronavirus 2 (SARS-CoV-2), the virus responsible for the COVID-19 pandemic that began in March 2020, has led to over 776 million confirmed cases worldwide, with an increase of more than 84,000/week in recent weeks. In the United States alone, over 103 million infections have been reported [1]. The virus has resulted in over 7.06 million deaths globally, with a significant portion—over 1.2 million—occurring in the United States [1]. Furthermore, a meta-analysis of studies conducted through 2022 indicates that Long COVID-related symptoms affect approximately 43% of COVID-19 subjects worldwide [2]. Based on this meta-analysis and Centers for Disease Control and Prevention data, LC’s prevalence ranges from 20% to 31% in the United States and is perhaps similarly prevalent worldwide [2,3].

Long COVID (LC; *aka*, post-acute sequelae of SARS-CoV-2 infection (PASC), post-COVID-19, post-acute COVID-19 syndrome, or long-haul COVID) [4,5,6,7], is a multi-organ condition characterized by persistent symptoms in individuals following infection with SARS-CoV-2. More specifically, it is defined as an ongoing pattern of relapsing and remitting changes in the functionality of multiple systems over time. Some definitions of this condition include any new symptoms or medical conditions lasting 30 days or longer following active SARS-CoV-2 infection [8]. Despite intensive efforts, there is still no universally accepted case definition for LC. According to the World Health Organization (WHO) Delphi Consensus case definition, LC is identified in individuals with a history of probable or confirmed SARS-CoV-2 infection who continue to experience a variety of symptoms or impairments, such as cognitive dysfunction, fatigue, shortness of breath or others, for nearly three months or more without an alternative diagnosis to explain these symptoms following the onset of the original acute disease [9].

LC can occur regardless of an individual’s vaccination status and is likely to occur even in cases of reinfection with SARS-CoV-2 [10]. The condition may persist for several years, increasing costs for the private and public health systems for those affected [11,12,13,14,15,16]. Individuals with LC also face negative impacts on their health-related quality of life, which can lead to loss of income [17,18] and rising personal healthcare expenses [19,20,21].

This manuscript offers a narrative review of the shared population characteristics between periodontitis and LC. We also describe the tissue, immunoinflammatory, and microbiologic events initiated by the initial SARS-CoV-2 infection and how they progress to LC. The short- and long-term impacts of SARS-CoV-2 on each cell or component of the immune system, along with their links to the pathogenesis of periodontitis, will also be examined. We aimed to determine if LC has plausible biological pathways and is worth epidemiological investigation to establish its association with periodontitis.

## 2. Methodology of the Literature Search

To provide a comprehensive overview of Long COVID (LC) and its potential interactions with periodontal and oral health, a broad literature search was conducted in PubMed. Search terms included both MeSH and natural-language keywords related to LC (e.g., “long COVID,” “post-acute COVID-19 syndrome,” “post-acute sequelae of SARS-CoV-2 infection,” “PASC,” “long-haul COVID”) and periodontal/oral diseases (e.g., “periodontal disease,” “periodontitis,” “gingivitis,” “oral disease,” “dental”). Searches were restricted to English-language publications with free full-text access, from January 2019 through 30 December 2024.

Initial queries identified 12,748 LC-related articles and 122,070 articles related to periodontal or oral health. The combined search terms identified 260 articles specifically addressing both LC and oral health, which were further screened manually to ensure relevance.

Selected studies initially focused on COVID-19 and LC pathophysiology, immune dysregulation, oral and periodontal manifestations, microbiome alterations, endothelial dysfunction, and SARS-CoV-2 persistence in oral tissues. Additional targeted searches were subsequently performed for specific topics, including biological mechanisms, pathogenesis, epidemiology, and clinical outcomes, to capture the most pertinent and up-to-date evidence. Through this meticulous, multi-step approach, approximately 248 articles were ultimately included in this review, representing a comprehensive synthesis of current knowledge on the intersections of LC and oral health.

## 3. LC Reported Symptoms and Clinical Manifestations

LC encompasses a broad spectrum of physical, psychological, and cognitive symptoms. These include fatigue, malaise, muscle pain, dyspnea, memory loss, hair loss, and migraines, as well as attention and sleep disorders. LC can lead to adverse clinical manifestations across multiple body systems, including but not limited to the respiratory, neurological, cardiovascular, gastrointestinal, metabolic, renal, and reproductive systems [5,15,16,22,23,24,25,26]. The disease is typically more pronounced in individuals with weakened immune systems or those who are immunocompromised [27].

## 4. LC Clinical Manifestations in the Oral Cavity

Although numerous reports have documented oral complications associated with SARS-CoV-2 [28], few have addressed oral manifestations of LC. The symptoms and clinical manifestations of LC may include taste disorders (e.g., hypogeusia or ageusia), chronic oral dysesthesia, ulceration, discoloration, hemorrhagic changes in the oral mucosa, aphthous-like lesions, atrophic cheilitis, alterations in salivary glands, tooth mobility and dental abscess, oral mucormycosis, and actinomycotic osteomyelitis [29,30,31,32,33,34,35]. To the best of our knowledge, the potential effects of LC on periodontitis—the most common oral disease after dental caries [36]—have been explored only minimally. However, there is significant potential for a strong relationship between these two conditions, given their shared chronic immune and inflammatory status. One cross-sectional study, which used a limited definition of LC, reported a higher prevalence of “sequelae from COVID-19” in individuals with periodontitis and obesity compared to those with obesity alone [37].

## 5. Periodontitis: Definition and Systemic Impact

Periodontal diseases affect the tissues that protect and support teeth. Most are induced by dental plaque and can be categorized into gingivitis, a periodontal tissue inflammation without loss of attachment or alveolar bone support, and periodontitis. Gingivitis is a reversible response to supragingival dental plaque and has a universal prevalence [38]. In contrast, periodontitis is a chronic condition that results in the loss of attachment and/or alveolar bone, with a prevalence of 42.2% in the U.S. [39]. Severe periodontitis often results in tooth loss and impacts approximately 7.8% of the U.S. adult population aged 30 and older [39] and 11.2% globally [40]. It contributes to a poor quality of life and is the most common form of bone pathology in humans [41].

Periodontitis has been linked to various systemic conditions, a subfield termed ‘periodontal medicine’, which explores two primary avenues: (A) the contribution of periodontitis to diseases in other parts of the body, facilitated by bacterial migration and the influence of active periodontitis on systemic inflammation, as evidenced by its association with coronary heart disease [42,43]; and (B) how systemic conditions, such as diabetes, influence the immune system and potentially exacerbate periodontitis. Notably, diabetes is recognized as a significant risk factor for periodontitis [44,45,46,47]. Therefore, examining the mechanisms of LC function and their potential impact on periodontal immunity, leading to subgingival dysbiosis and subsequent periodontal damage, forms a hypothesis modeled on the relationship between diabetes and periodontitis.

While it can be argued that there are differences in systemic and local immune profiles during infections, it is important to note that advanced periodontitis leads to a destructive immune response both locally (gingivally) and systemically [48,49], worsening any existing systemic inflammation [42,43]. It might intensify the initial COVID-19 infection or raise the risk of leukocyte complications [50]. Conversely, the immunoinflammatory dysregulation associated with LC could trigger the onset, progression, and severity of periodontitis, similar to diabetes [51,52].

## 6. Epidemiologic Factors Shared Between LC and Periodontitis

LC has been reported to occur in individuals exhibiting specific characteristics, such as smokers, the elderly, women, a lower socio-economic status (SES), ethnic minorities, a history of severe symptoms during acute COVID-19, pre-existing psychiatric disorders, and pre-existing chronic comorbidities such as obesity, diabetes, asthma, cardiovascular disease (CVD), and hypertension [2,6,53,54]. Periodontitis shares many of these characteristics, like older age, low SES, and a higher prevalence among ethnic minorities [55,56]. Additionally, periodontitis often coexists with conditions prevalent in the LC group, such as chronic mental health disorders and cardiometabolic diseases (e.g., diabetes, CVD, and obesity) [57,58,59]. Specific population subgroups also exhibit distinct characteristics related to periodontitis, such as low educational attainment, infrequent dental visits, and poor dietary habits [55,56,60,61].

## 7. Immuno-Pathophysiology Mechanisms Shared by LC and Periodontitis

### 7.1. Early Immune Response to the SARS-CoV-2

#### 7.1.1. Interferon-Mediated Local Innate Immune Response to a Viral Infection

Under normal conditions, innate immunity tracks a generic viral infection to its source and combats it. This process is mediated by type I interferon (IFN) and related molecules and occurs within a couple of hours [62]. It limits viral replication within infected cells, slows the virus’s spread, and activates the adaptive immune response, which involves the simultaneous actions of B cells generating neutralizing antibodies alongside T cells, including CD4+ and CD8+, which quickly eliminate infected cells and their viral components [62,63,64].

However, during acute SARS-CoV-2 (or initial) infection, this virus can effectively suppress [65] or evade activation of the immune system through type I and type III IFN responses, allowing it to continue replicating [66,67]. Additionally, the adaptive immune responses are similarly impaired, as the innate immune system cannot quickly present antigenic information [63]. Along with the impaired or delayed type I and type III IFN innate immune regulation [65,68], low dendritic cell counts, a deficit in IFN-α expression [69,70], and a reduction in the total number of NK cells are observed [71], along with the depletion and exhaustion of T cells [72].

Dendritic cells (DCs) are the main source of type I IFN in gingival tissue, with higher levels seen in subjects with periodontitis [73]. However, in the complex etiologic model of periodontitis, where bacterial and viral challenges can occur simultaneously, a decrease in type I IFN and IFN-γ occurs due to co-infection with organisms from these two groups [74]. Studies on the link between Herpesviruses and periodontitis have also shown lower IFN levels in periodontitis patients who tested positive for these viruses [75] or during active periodontitis [76]. It seems that periodontal infection by Herpesviruses and SARS-CoV-2 may use similar strategies to evade or reduce IFN expression by dendritic cells.

#### 7.1.2. The Short-Term T-Cell Response to SARS-CoV-2

Among SARS-CoV-2-positive asymptomatic individuals, delays in innate immune responses do not prevent long-term viral clearance. The adaptive immune responses, which include increased CD4+ and CD8+ T-cell counts and higher levels of neutralizing antibodies, effectively compensate for the impaired innate response. These antibodies are produced more quickly than usual to fight the viral infection [77]. The presence of active T cells in infected tissues and antibodies made by B cells against SARS-CoV-2 indicates disease resolution [63,78,79].

Among patients with acute COVID, there is functional impairment of adaptive immune responses, characterized by decreased total B cells, CD4+ T cells, and CD8+ T cells (lymphopenia). It is often accompanied by increased survival but functionally exhausted T cells [63,64,72,79,80]. Immature neutrophils particularly disrupt T-cell polarization, promoting Th17 cell differentiation while suppressing Th1. This causes major disturbances in the immune response against SARS-CoV-2 [81,82,83]. Key Th17 effector cytokines, including members of the IL-17 family (primarily IL-17A and IL-22), play crucial roles in the pathogenesis of COVID-19 and periodontitis [79,84,85,86,87].

Antigen persistence, antibody production, and T-cell counts are closely linked. The stability of CD4 and CD8 T-cell responses is one of the findings related to recovery from acute COVID-19 [88,89], with fewer tissue cells presenting T-cell receptors [90]. However, antibody titers against SARS-CoV-2 may vary between individuals, showing both reductions and partial persistence [91], as well as somatic evolution effects [92].

The role of CD8+ T cells in chronic periodontitis is less prominent. CD8+ T cells seem to help preserve alveolar bone by suppressing osteoclastogenesis [93,94]. However, their function can be overlooked due to the higher levels of other immunoinflammatory responses triggered by periodontal microbial infections, which significantly disrupt bone homeostasis. The overreactive immune response under LC may hide the role of CD8+ cells even more [95].

#### 7.1.3. Neutrophils and the “Cytokine Storm”

The immune system uses various strategies to compensate for impairments caused by severe SARS-CoV-2 infection, including an increase in neutrophil count [96] as early as the first week [97,98]. Neutrophil activation boosts inflammation, promotes prothrombotic cycles, and causes persistent production of pro-inflammatory cytokines such as Interleukins (IL)-1β, IL-2, IL-6, IL-7, IL-8, IL-10, IL-17, IL-21, IL-23, granulocyte colony-stimulating factor (G-CSF), monocyte chemoattractant protein 1 (MCP-1), and tumor necrosis factor (TNF), collectively known as the ‘cytokine storm’ [64,71,98,99]. The continued presence of high neutrophil counts and abnormal responses after the acute phase of COVID serves as a marker of severe LC [100,101,102]. In the periodontium, the pathogenic pathways underlying plaque-induced periodontal diseases involve many cytokines from the storm, which play important roles [103,104]. IL-1β, IL-6, IL-17, IL-23, and TNF are well documented in periodontitis, having various roles in immune cell recruitment, pro-inflammatory activity, and periodontal tissue destruction. Conversely, IL-2 and IL-10 promote less inflammation and tissue damage.

Neutrophil-extracellular traps (NETs) are another mechanism that enhances inflammation and promotes blood clotting from these cells [105], in addition to the NETs’ primary role as traps for pathogens [105]. NETs consist of the extracellular release of chromatin combined with histones, proteases, lactoferrin, cathepsins, and myeloperoxidase [106], and are associated with several chronic diseases, especially chronic obstructive pulmonary disease (COPD) and asthma [107]. Several studies have detected NETs in the blood of severe COVID-19 patients [108,109,110].

Circulating NETs, identified by cell-free DNA (cfDNA) or myeloperoxidase (MPO)-DNA in blood, are standard markers of acute diseases, including COVID, and are also found in LC [111]. However, cfDNA or MPO-DNA alone is not an accurate NET marker, as it also indicates cell necrosis and apoptosis [112]. The most effective way to use circulating cfDNA/MPO-DNA as a NET marker is in combination with tissue biopsies. Despite this, it is suggested that the initial SARS-CoV-2 infection may cause epigenetic changes in parenchymal, vascular, and immune cells, including myeloid progenitor cells in the bone marrow, leading to neutrophil formation and NETs [111]. These hypotheses remain unconfirmed.

NETs are recognized as key components of the immune response against plaque-induced periodontal diseases [113], particularly in the acute phases of inflammation. Biopsies show higher NET expression in gingivitis than in periodontitis [114]. Both periodontitis and severe COVID share elevated cfDNA/MPO-DNA levels associated with NET-mediated inflammatory responses. MPO-DNA levels positively correlate with probing depths and clinical attachment loss, with the highest levels seen in individuals with periodontitis and rheumatoid arthritis [115]. This similarity has been suggested as a potential reason why periodontitis could exacerbate the initial COVID-19 infection [106].

#### 7.1.4. Monocytes and Macrophages

Another key component of the immune response to SARS-CoV-2 that plays a significant role in periodontitis is monocytes and macrophages, which have been reported to be hyperactivated and dysregulated in relation to severe COVID [116,117]. Monocytes and macrophages are involved in virus clearance; however, their dysregulation worsens tissue damage [117,118,119]. Similar to neutrophils, when activated, these cells produce cytokines and undergo pyroptosis—inflammation-induced cell death [120,121].

Key inflammatory molecules expressed by these two cells when dysregulated are IL-1α, IL-1β, IL-6, IL-7, THF, IFNs I and II, CCL2, CCL3, and CXCL10 [117], a molecular profile linked to hyperinflammation and severe diseases [118,122]. Specifically, elevated systemic IL-6 levels prompted the use of inhibitors of this cytokine as therapeutic drugs against COVID, such as tocilizumab and siltuximab [111].

Hyperactivation of monocytes and macrophages has been reported in LC [123], potentially causing ongoing tissue damage. Active monocytes have been detected from eight to fifteen months [124] of LC, regardless of the severity of the initial or acute infection. Persistent monocyte/macrophage activation has been demonstrated in the lungs of individuals with PASC/LC [125], and mechanistic studies support ongoing macrophage-driven inflammation as a contributor to LC pathophysiology [126]. In vitro assays with macrophages from subjects recovered from COVID have demonstrated persistent hyperregulation of inflammatory molecule production and downregulation of pro-resolving factors [127].

Hyperactive monocytes and macrophages are part of the inflammatory response in periodontitis [128,129]. This cell is a key finding in the hypothetical pathway linking periodontitis to cardiovascular diseases [130], suggesting that hyperreactive macrophages from active periodontitis sites could enter the bloodstream, causing endothelial damage or affecting other organs. Therefore, in patients with periodontitis infected with SARS-CoV-2, a pro-inflammatory monocyte/macrophage profile is already present. Conversely, the persistence of this pro-inflammatory profile can exacerbate the response to dental plaque challenge, leading to periodontal destruction.

IL-6 is a crucial cytokine in periodontitis. Elevated levels in the gingival crevicular fluid (GCF) are linked to alveolar bone loss and dysbiotic biofilms [131,132]. However, in an animal model, IL-6 increases from gingival epithelial cells during common daily activities, such as chewing [133], in response to mechanical damage. This IL-6-driven process primes resident TH17 cells, serving as a key defense mechanism of that specific mucosal barrier, regardless of pathogen presence.

Elevated IL-6 is a pathway that links periodontitis with diabetes [134], coronary heart disease [135], and COPD [136]. Pre-existing circulating levels of IL-6 before and after the initial COVID infection, owing to its pro-inflammatory effects and regulation of dendritic, B, and T cells [137], would favor LC. Conversely, LC may act as the initial trigger for systemic IL-6 elevation, leading to damage in various tissues, including the periodontium.

**Figure 1:**
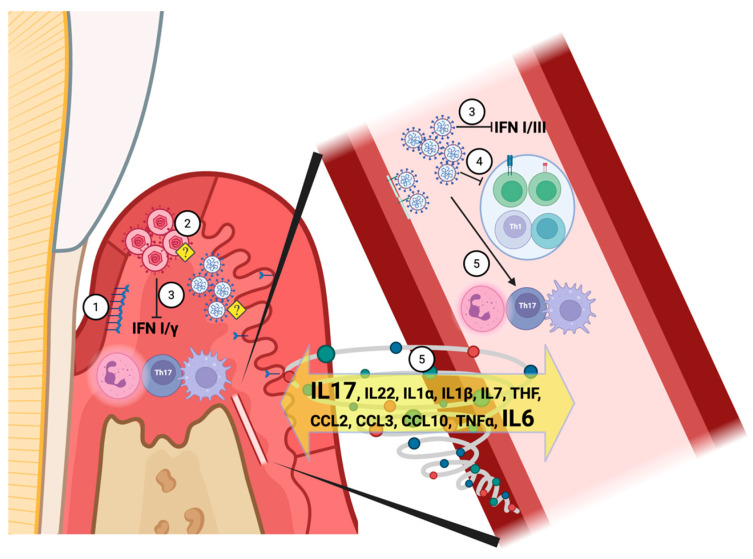
Overlapping or concomitant mechanisms between Initial COVID and Periodontitis. Created in BioRender. Mattos, M. (2025). Overlapping or concomitant mechanisms between Initial COVID and Periodontitis. (1) ACE2 and TMPRSS2 are expressed in the gingival epithelium, with higher expression during inflammation. (2) Gingival invasion and SARS-CoV-2 replication could also trigger replication of herpesviruses, most notably Human CMV and Epstein–Barr Viruses. (3) The herpesviral replication process in the gingiva involves inhibition of Interferon-1 and Gamma; similarly, SARS-CoV-2 has been shown to inhibit Interferon-1 and 3 in infected tissues. (4) SARS-CoV-2 infection reduces the numbers and activity of CD4 and CD8 T cells, as well as T-Helper 1 and B cells, while increasing T-Helper 17, neutrophils, and macrophages/monocytes as a compensatory response, leading to the (5) cytokine storm, characterized by a significant production of Interleukins 6 and 17. <?> Denotes a putative mechanism, requiring scientific validation (Created in BioRender. Mattos, M. (2025)—https://app.biorender.com/illustrations/687e854e999482dcdee06cc7?slideId=d9cb3364-c854-4483-ae0b-4a1ece176454).

As illustrated in Figure 1, these overlapping mechanisms between initial COVID-19 and periodontitis involve interconnected viral, immune, and inflammatory pathways.

### 7.2. A Persistent Active Immune Response to the SARS-CoV-2, the Key to LC

#### 7.2.1. The Resolution of the SARS-CoV-2 Infection

Recovery from COVID-19 is common; however, an altered immune phenotype and/or function may persist in convalescent individuals [138,139,140]. Neutralizing antibody levels decline steadily with the severity of the initial acute COVID-19 infection. Individuals with a high peak infectious viral load have maintained elevated neutralizing antibody titers afterward. In contrast, those with lower loads experience a gradual decline, returning to baseline levels even more than three months after disease onset [141].

An example of a persistent immune response is seen in elderly individuals who survive severe COVID-19 and show a late increase in circulating CD4+, CD8+, and double-negative B cell populations. Over time, this is marked by improvements in dysfunctional T and B cells (including changes in cell memory) and increased levels of cell activation and exhaustion, which contrasts with the depletion seen during the acute phase [64,70,142,143,144].

LC is linked to ongoing immunological dysregulation and can affect individuals from initially asymptomatic to severe cases [142,145,146,147,148], with continued neutrophil activity, persistent production of inflammatory cytokines, and deficient naive T- and B-cell counts [142,143]. Periodontitis, in alignment with the continuous activity of neutrophils and persistent inflammatory cytokine production observed in LC, is characterized by an imbalanced and dysregulated recruitment of neutrophils due to overwhelming microbial challenges [95,149].

However, the dysregulation seen in periodontitis opposes the reduction in cells observed in LC for T cells, B cells, natural killer (NK) cells, macrophages, and DCs, all of which contribute to the start and worsening of periodontitis [149,150,151], thereby further boosting pro-inflammatory cytokine production.

#### 7.2.2. A Persistent Dysregulation of T Cells in LC

The behavior of T cells after the initial COVID infection is likely a key factor that contributes to LC. However, findings regarding these cells are inconsistent. In some cases, a short life cycle of cytotoxic CD8+ cells was observed [152], along with a stronger signal of cell death from T cells than from antigenic memory [153]. On the other hand, studies involving subjects with areas of persistent SARS-CoV-2 infection, such as the lungs, show larger numbers of circulating specific CD8+ T cells with increased production of IFN Gamma, TNF, IL-6, and C-reactive protein [154]. Similar patients have also shown dysregulation of tissue-resident CD8+ T cells from lung biopsies [155]. If the diseased periodontium is shown to be another area where persistent SARS-CoV-2 is detected, the existing cellular and cytokine profile in periodontitis will be similar and synergistic with LC.

#### 7.2.3. A Broad and Continuous Adaptive Immune Response Is Part of LC

Although most patients recovering from acute COVID will show a reduced count of antibody-secreting B cells during this stage, a small percentage will maintain elevated counts of these cells [156]. In line with this finding, LC is characterized by broad adaptive immune activation, with B cells resuming the production of antibodies against past infections, alongside an increase in CD4+ and CD8+ specific lineages targeting SARS-CoV-2 and cytomegalovirus during convalescence [157].

Another way the adaptive immune system sustains activity in LC is through COVID-driven autoimmunity. This phenomenon occurs even during the acute phase. It aims to neutralize IFN type I via autoantibodies against it [158,159,160], resulting in a reduced IFN response, failed SARS-CoV-2 clearance, and ongoing local and systemic inflammation [65,67,68,161,162]. Reports of autoantibodies against other cytokines, chemokines, cell-surface proteins [163], and phospholipids [164] have all been noted.

In LC, autoantibody production is continuous due to the still active adaptive immunity via double-negative B cells, even in subjects with mild initial COVID [156,165]. This pattern resembles several autoimmune diseases, as it involves autoantibodies against components of connective tissue, cytokines, chemokines, and antinuclear antibodies [166].

The role of autoimmunity in LC remains unclear. Studies vary, with some demonstrating a correlation between autoantibodies and LC [167], while others do not [168]. Opinions differ on whether autoimmunity is a risk factor for LC [157], if LC is an autoimmune disease, or if autoantibodies could offer protection against LC [169]. There is no consensus on the issue.

### 7.3. The Complement System

The complement system, an integral part of innate immune regulation that plays a crucial role in immunity and homeostasis by targeting pathogens and damaged cells, has recently been implicated in the activation mechanisms of L [170]. Individuals with LC exhibit an imbalance in terminal complement complex (TCC) formation, which is characterized by elevated levels of soluble C5bC6 complexes, diminished concentrations of C7-containing TCC formations, and associated thrombo-inflammation. Current literature highlights increased markers of tissue injury, red blood cell lysis, platelet activation, and monocyte–platelet aggregates [170]. Active LC is characterized by sustained activation of specific alternative and classical complement pathways.

The complement system might be involved in periodontitis’ pathogenesis as well [171]. Complement proteins are activated in high quantities during active periodontitis, with their components and cleavage products found in the diseased gingival tissues, in contrast to being undetected or present in low levels in healthy gingival tissues. The complement components identified in the affected gingiva or GCF encompass the entire immune cascade, including C1q, factor B, Bb, C3, C3a, C3b, C3c, C3d, C4, C5, C5a, C5b, and C9. A single nucleotide polymorphism in the gene coding for complement C5 and C3 has been linked to periodontitis [172]. C3a and C5a, along with mast cells, are involved in osteoclastogenesis and alveolar bone loss [150].

The key immune–inflammatory components shared between LC and periodontitis are summarized in Table 1, highlighting similarities and differences in immune cell activity, cytokine production, and other mediators.

## 8. LC Inflammation at the Tissue Level

### 8.1. The Role of IL-17, RANKL, and Matrix Metalloproteinases (MMPs) in Bone Metabolism Related to LC

In LC, SARS-CoV-2 may also directly affect bone cells through angiotensin-converting enzyme 2 (ACE2) receptors expressed on osteoclasts and osteoblasts, potentially disrupting the bone remodeling process [173,174]. Heightened production of inflammatory cytokines, recruitment of Th17 cells, and alterations in RANKL and osteoprotegerin (OPG) signaling could represent proposed mechanisms linking COVID-19 to osteoporosis and bone loss [174,175]

Animal studies provide additional mechanistic support. Cytokine storms characterized by elevated IL-1β, IL-6, and TNF-α induce trabecular bone loss in long bones and lumbar vertebrae, worsening from the acute to post-recovery phase in SARS-CoV-2-infected hamsters [176]. Th17 cells promote osteoclastogenesis through IL-17-mediated induction of RANKL [177], and elevated Th17 cells or IL-17 may contribute to bone-related outcomes in LC [175].

Consequently, elevated inflammatory markers in LC are associated with increased expression of MMPs, including MMP-1, 2, 3, 7, 8, and 9, which have been linked to pathophysiological processes ranging from chronic fatigue symptoms to rapid progression of pulmonary fibrosis [178,179,180,181,182]. Specifically, MMP-1, 8, and 9 are significantly elevated and correlate with disease severity, neutrophil degranulation, endothelial dysfunction, metabolic dysregulation, and pulmonary fibrosis [178,179,180,181,182]. MMP-8, primarily secreted by neutrophils, is a major enzyme that degrades interstitial collagens in periodontitis [183,184]. Additionally, MMP-8 and MMP-9 contribute to COVID-19 severity [185,186,187].

At the level of periodontal disease sites, CD4+ T cells (which are systemically decreased in LC) in the gingival tissue are the primary contributors to increased receptor activator of RANKL levels in individuals with chronic periodontitis [151]. However, neutrophils can also promote the progression of periodontitis by inducing the recruitment of Th17 cells (systemically elevated in LC), which are later activated and differentiated by dendritic cells (DCs) [188,189] in active periodontitis [190], and by driving the accumulation of B cells and plasma cells in severe lesions [95].

The IL-17 cytokine-derived Th17 cells produced in periodontal tissue [191,192] can stimulate macrophages and other tissue sources, such as endothelial cells, epithelial cells, and fibroblasts, to locally produce pro-inflammatory mediators (e.g., IL-6, IL-8, TNF-α, IL-1β, and PGE2), with partial synergy with LC’s cytokine storm [95,183] and can direct osteoblasts to produce RANKL [193] and promote bone resorption [183,194,195].

B cells’ local production of neutralizing antibodies may directly or indirectly contribute to the destruction of periodontal connective tissue or alveolar bone by producing pro-inflammatory cytokines, MMPs, and RANKL [95,196,197].

### 8.2. The Role of Angiotensin-Converting Enzyme 2 (ACE2) on the LC and Periodontitis Association

Another biological pathway linking LC to periodontitis involves the role of ACE2 receptors, which are abundant in various human tissues [198]. ACE2 is the primary receptor for SARS-CoV-2 entry and invasion of several organs and tissues, mainly through the co-receptor “Transmembrane protease serine 2” (TMPRSS2) in the upper respiratory epithelium [199,200,201,202]. After initial infection, SARS-CoV-2 causes cells to overexpress ACE2 via 1) direct binding of its spike protein to the ACE2 receptor [203] or (as recently reported) 2) direct or indirect binding and activation of Toll-like receptor 4 (TLR4) [204], followed by fusion of the viral and host cell membranes, facilitated by TMPRSS2 and furin molecules [203,205,206], leading to replication, worsening of the infection, and ongoing inflammation [204].

Both ACE2 and TMPRSS2 are found in the epithelial part of the gingiva, specifically in the sulcular and pocket epithelia. ACE2 is located in the nucleus and cytoplasm of the spinous-basal layer, while TMPRSS2 is present in the gingival epithelium as well as the horny and spinous-basal layers, with its expression in the cytoplasm and cell membrane [207].

Prior or ongoing infections of mucosal, epithelial, and endothelial cells—associated with inflammation in the respiratory, renal, vascular, gastrointestinal, or cardiac tissues—raise the expression of ACE2, making these tissues more vulnerable to SARS-CoV-2 infection [208]. SARS-CoV-2 infections of the endothelium introduce the virus to various target organs [209], framing COVID-19 as a vascular disease [210].

Cytokine-mediated tissue stimulation from COVID-19 or other inflammatory processes can increase ACE2 expression, potentially creating a positive feedback loop that enhances viral replication [211,212,213,214]. Therefore, prolonged or latent chronic SARS-CoV-2 antigen exposure from ACE2-rich tissues could trigger the immunological dysregulation seen in LC [142,215].

Higher titers of plasma ACE2 activity have been observed in individuals with LC for up to four months following acute SARS-CoV-2 infection [215]. Similarly, SARS-CoV-2 RNA and/or proteins have been found long after the initial infection in gastrointestinal tissues, including the stomach, colon, intestine, appendix, gut mucosa, epithelium, and colorectal lamina propria, as well as in other tissues such as skin, breasts, gallbladder, and olfactory neuroepithelium.

Additionally, SARS-CoV-2 viral particles have been detected in the stool and blood of LC patients [216]. The presence of viral RNA in the plasma [157] and the persistence of the virus in “immunologic sanctuaries” like the gastrointestinal tract, olfactory system, and brain [70,217] are considered factors that may predict LC.

However, when long-term SARS-CoV-2 antigen shedding is the main variable in cross-sectional epidemiologic studies, the links with LC vary by location. In those studies, no association was found between salivary SARS-CoV-2 long-term shedding and LC [152] or persistent gastrointestinal disease [217]. Only a link was seen between SARS-CoV-2 in the olfactory mucosa and local LC symptoms [218].

Evidence of SARS-CoV-2 presence in the oral cavity during LC is still limited. SARS-CoV-2 was found in subsequent tissue biopsies of fungiform papillae from patients with LC sequelae [219]. SARS-CoV-2 has been detected in the supragingival and subgingival biofilms of individuals with acute COVID-19, even those without periodontitis [220], and in the saliva of individuals with asymptomatic or mild COVID-19 [221]. Similarly, ACE2 activity in saliva correlates with an individual’s susceptibility to SARS-CoV-2 infection and disease severity [222]. The epithelial cells of the oral mucosa, tongue, and salivary glands exhibit higher ACE2 expression; these cells may serve as initial and late sites for SARS-CoV-2 infection [50,223].

Furthermore, similar to other viral infections, such as herpesviruses [224,225], periodontal pockets with ulcerated gingival epithelium, exposed connective tissue, and periodontal ligament cells that express higher levels of ACE2 [207] could serve as reservoirs or immunologic sanctuaries for SARS-CoV-2 [226]. The presence of the virus in periodontal pockets could worsen infections at both the periodontal and systemic levels.

## 9. The Microbiologic Impact of LC

### 9.1. The Reactivation of Viral Infections as a Result of LC Immune Response

Other viral infections may re-emerge during COVID-19, with the Epstein–Barr virus (EBV) being well documented in the literature. This co-infection is considered a potential factor in LC and is associated with neurocognitive symptoms such as fatigue [227].

EBV is one of the herpesviruses associated with periodontitis, similar to cytomegalovirus. Traditionally, EBV is an etiologic factor for Hairy Leukoplakia, classically regarded as a pathognomonic indicator of HIV infection, but it is not limited to this [228] and is also associated with some oral cancers [229]. EBV is linked to periodontitis in populations across Asia, Europe, and America. It can be detected in subgingival plaque and GCF, and a positive correlation has been observed between EBV presence, gingival inflammation, and probing depths [230]. Active periodontitis can serve as a reservoir for EBV, which may co-infect with SARS-CoV-2.

The co-infection of EBV with SARS-CoV-2 may also contribute to the autoimmunity linked to LC, supported by scientific evidence showing that EBV alone can cause autoimmunity [231,232,233]. However, there is no agreement on whether COVID triggers autoimmune mechanisms or if these are genetically determined before the initial SARS-CoV-2 infection [158]. Furthermore, the ongoing activity of adaptive immunity does not necessarily mean that the autoimmune process will persist. For example, autoantibodies against IFN-1 are not associated with LC [234].

### 9.2. LC May Affect Oral Dysbiosis, Increasing Susceptibility to Chronic Infections, Including Periodontitis

Persistent immune dysregulation in LC may contribute to altered host responses, including susceptibility to disruptions in microbial homeostasis. Gut dysbiosis and long-term gastrointestinal complications have been described in individuals following acute SARS-CoV-2 infection or in those with LC [235,236,237]. SARS-CoV-2 RNA has been detected in stool samples months after the acute infection, and persistent fecal shedding has been associated with gastrointestinal symptoms in some individuals with LC [238,239]. These findings, together with reports of gut viral antigen persistence in post-acute COVID-19 [238], suggest that prolonged gastrointestinal involvement may occur in a subset of affected individuals.

Beyond the gastrointestinal tract, the posterior oropharyngeal microbiome may also be altered in COVID-19. A recent study demonstrated that COVID-19 can induce oropharyngeal dysbiosis that persists for at least 30 days, independent of viral clearance or antibiotic exposure [240]. Such persistent microbial shifts raise the possibility that the oropharynx may serve as a transient reservoir for opportunistic bacteria associated with respiratory or chronic lung infections. The relationship between periodontal pathogens and LC remains unclear. Bemquerer et al. (2024) found no significant changes in the composition of subgingival pathogens when evaluating periodontitis in individuals with acute COVID-19 [241]. In contrast, Haran et al. (2021) observed that higher early levels of Prevotella and Veillonella in acute COVID-19 patients were associated with prolonged symptoms and the eventual development of LC [242]. However, no studies to date have assessed whether chronic LC itself alters subgingival microbial symbiosis in ways that may contribute to periodontitis development.

## 10. The Potential Mechanisms of COVID-19/LC: Effects on Periodontitis

A mechanistic understanding of how LC might influence the development or progression of periodontitis is needed. A recent scoping review suggests that periodontitis and COVID-19 may independently raise serum levels of IL-1β, IL-6, and TNF-α in the same individual [243].

However, information on the combined or causal effects between the two diseases is lacking [243]. As previously mentioned, the link between ‘sequelae’ from COVID-19 and periodontitis has been evaluated in a cross-sectional study [37]. A reverse assessment in a case–control study of COVID-19 showed increased salivary IL-6 measured at two time points in individuals with both COVID-19 and periodontitis, while periodontitis was associated with elevated salivary levels of RANKL and IL-1β [241].

Another study showed that individuals with acute COVID-19 had a subsequent increase in the serum RANKL/OPG ratio compared with healthy controls. It also found that in vivo murine coronavirus (MHV-3), a SARS-like virus, can cause an osteoporotic phenotype through TNF and infect macrophages and osteoclasts in a mouse model of SARS-like disease [244].

**Figure 2:**
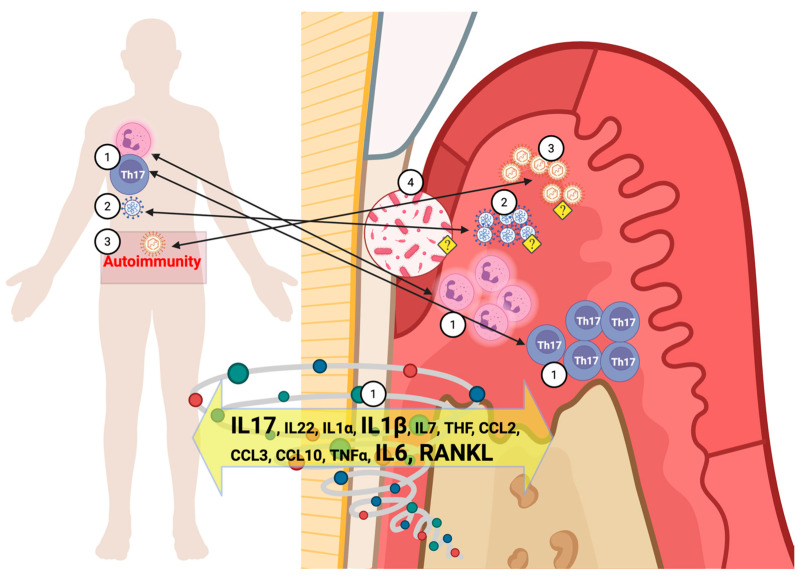
Shared pathways through which Long COVID (LC) can be a systemic risk factor for Periodontitis. Created in BioRender, Mattos, M. (2025). Pathways through which Long COVID (LC) can act as a systemic risk factor for periodontitis, and vice versa: (1) A persistent cell-mediated inflammatory response from LC, along with the cytokine storm, can increase the activity of neutrophils and T-helper 17 cells at sites with chronic infections, such as periodontitis. Markers of periodontal tissue destruction, such as RANKL, are elevated in LC subjects. (2) Similar to the lungs or gut, periodontal pockets can serve as reservoirs for SARS-CoV-2 and sources of reinfection by this virus. (3) As Epstein–Barr virus replicates, it may play a role as a potential cause of the autoimmunity observed in LC. (4) The persistent inflammation from LC and Periodontitis, along with the possible periodontal presence and replication of SARS-CoV-2 can be drivers for subgingival dysbiosis. <?> Denotes a putative mechanism, requiring scientific validation. (Created in BioRender. Mattos, M. (2025)—https://app.biorender.com/illustrations/6888ec8ebdbd2078a1d438e7?slideId=5c312c1d-e8f6-42d8-a5ed-39414e4499fe).

Figure 2 illustrates the possible bidirectional pathways connecting LC and periodontitis, emphasizing chronic inflammatory feedback loops.

## 11. Discussion

LC, an emerging disease with less than a decade of clinical reports, can persist after the acute phase of COVID-19. Similar to diabetes, the immunoinflammatory dysregulation associated with LC may increase susceptibility to periodontitis, making individuals with this condition a previously overlooked group for preventing and treating periodontitis. Although the epidemiological link between these two chronic conditions needs further investigation, the pathogenic pathways of both diseases share similarities and connections, as described throughout this manuscript, thus providing biological plausibility to any epidemiological studies of this association.

Biologic plausibility can be overlooked as a prerequisite for epidemiologic studies that explore the systemic relationships between periodontitis, dental health, and oral hygiene, which may be subject to criticism [245] and examples of spurious associations [246]. Given the biological plausibility between LC and periodontitis, observational studies will be necessary to confirm the association and its nature. The novelty LC offers is its additional pathogenic impact on individuals with existing chronic diseases, including periodontitis.

LC exerts a widespread pro-inflammatory effect on the immune system, marked by persistent increases in neutrophils, production of pro-inflammatory cytokines, and activation of immune cells, along with elevated levels of RANKL, MMPs, and complement factors—features common in both periodontitis and LC.

LC can lead to gut dysbiosis and may also cause subgingival microbiome dysbiosis, increasing the risk of periodontitis. Although evidence of SARS-CoV-2 presence in the oral cavity related to LC is limited, gingival and periodontal ligament cells in periodontal pockets exhibit increased ACE2 expression, indicating that areas with active periodontitis could serve as reservoirs for SARS-CoV-2. Nevertheless, further epidemiological, tissue, cell, and molecular studies are needed to confirm the relationship within the population and clarify the mechanisms linking LC and periodontitis.

It is plausible that the onset of periodontitis in previously healthy individuals or the worsening of existing periodontitis in those with a history may be an overlooked clinical sign of LC.

Therefore, epidemiologic and clinical investigations are crucial to deepen our understanding of the relationship between LC and periodontitis. Clarifying the connections between pre-existing chronic conditions (such as diabetes, cardiovascular disease, and obesity), their links with COVID-19 or LC, and periodontitis can provide valuable insights. Developing a comprehensive understanding of how LC interfaces with broader risk assessments, causal pathways, and syndemic interactions, as reflected in global burden frameworks, may provide dual benefits for improving both oral and systemic health in individuals with LC [247]. Ultimately, once a clear understanding of the LC-periodontitis relationship is established, preventive efforts and policies can be developed to raise awareness of the potential development or worsening of periodontitis and related systemic complications in the affected population, including those with cardiometabolic cardiovascular disease [248].

## Figures and Tables

**Table 1:** Summary of expression patterns of inflammatory and immune components in LC and periodontitis.

Immune—Inflammatory Components	Long-COVID	Periodontitis
Dendritic Cells	↓	↑
IFN-α Production	↓	↑ or ↓
Neutrophils	↑	↑
Pro-Inflammatory Cytokines and other Inflammatory mediator productions—“Cytokine Storm”	↑ IL-1β, IL-2, IL-6, IL-7, IL-8, IL-10, IL-17, IL-21, IL-22, IL-23, G-CSF, MCP-1, TNF-α	↑ IL-1β, IL-6, IL-8, IL-10, IL-17, IL-21, IL-22, IL-23, PGE2, TNF-α
Natural Killer Cells	↓	↑
Complement Factors	↑ or ↓ ↑ Soluble C5bC6 complexes, ↓ C7	↑ ↑ C3, C3a, C5, C5a
Total Number of T and B Cells	↑ In the majority of affected: persistent immune exhaustion; maintained elevation (i.e., delayed or dysregulated recovery), or late in some individuals (e.g., the Elderly or Immunocompromised)	↑
Neutralizing Antibodies	Acute-phase viral-load dependent (e.g., elevated in individuals with a high peak infective viral load)	↑
CD4+ T Cells	↑ or ↓ ↓ In the majority of affected; ↑ Among the Elderly	↑
CD8+ T Cells	↑ or ↓ Inconsistent: ↓ In the majority; but maintained elevated or with late ↑ in some individuals (e.g., the Elderly or Immunocompromised)	Less noticeable in the literature
Th17	↑	↑
MMPs (e.g., MMP-8, MMP-9)	↑	↑
RANKL	↑	↑

Arrows indicate general trends: ↑ increase, ↓ decrease. Long-COVID responses may vary with age or pre-existing conditions. Abbreviations: IL = Interleukin; TNF = Tumor Necrosis Factor; G-CSF = Granulocyte Colony-Stimulating Factor; MCP = Monocyte Chemoattractant Protein; PGE2 = Protaglandin E2; C = Complement; MMP = Matrix Metalloproteinase; RANKL = Receptor Activator of Nuclear Factor kappa-B Ligand; IFN = Interferon; CD = Cluster of Differentiation.
